# High MAF of *EGFR* mutations and high ratio of T790M sensitizing mutations in ctDNA predict better third‐generation TKI outcomes

**DOI:** 10.1111/1759-7714.13418

**Published:** 2020-04-14

**Authors:** Yan Li, Fanshuang Zhang, Pei Yuan, Lei Guo, Ying Jianming, Jie He

**Affiliations:** ^1^ Department of Pathology and National Cancer Center/Cancer Hospital Chinese Academy of Medical Sciences and Peking Union Medical College Beijing China; ^2^ Thoracic Surgery, National Cancer Center/Cancer Hospital Chinese Academy of Medical Sciences and Peking Union Medical College Beijing China

**Keywords:** High‐throughput NGS, liquid biopsy, MAF, outcomes, ratio

## Abstract

**Background:**

Clinical detection of *EGFR*‐TKI resistance mechanism through tissue can be really challenging due to risks associated with the procedure. Thus, liquid biopsy, especially circulation tumor DNA (ctDNA) analysis, can be an adequate source for biomarker testing in targeted therapy. Our study was aimed at clinical validation of liquid biopsy next‐generation sequencing (NGS) by comparison with tissue biopsy, and we also investigated clinical utility of ctDNA NGS on the prediction of TKI outcomes.

**Methods:**

Using hybrid capture panel NGS, we compared the concordance, sensitivity, and specificity of ctDNA using 39 paired plasma and tissue biopsy, and investigated the association between ctDNA genomic alterations of 147 first‐generation TKI‐relapsed patients and their response to first‐ and third‐generation TKIs.

**Results:**

The concordance for ctDNA and tissue biopsy was 84.62% among all patients, and even higher among late stage patients (88.24%). Among 147 *EGFR*‐TKI‐relapsed patients, T790M was the most common reason for resistance (40.13%). Compared with T790M‐positive patients, patients only detected with sensitizing mutations (sensi‐mutations) had lower mutant allele frequency (MAF) of sensi‐mutations (*P* = 0.031). *TP53* mutation showed negative impact on TKI treatments. In survival analysis of third‐generation TKI, we found a positive correlation between ratio of T790M sensi‐mutation and PFS (*P* = 0.018); also, higher MAFs of both sensi‐mutation and T790M were observed in the PR group than the SD + PD group.

**Conclusions:**

Both ratio of T790M sensi‐mutations and MAFs of *EGFR* mutations were associated with third‐generation TKI outcomes. Thus, incorporation of high‐throughput NGS into clinical trials may be crucial to identifying the response to osimertinib, as it provides more comprehensive genomic information.

**Key points:**

High concordance of ctDNA and tissue biopsy was observed. NGS of ctDNA from 147 TKI‐relapsed patients showed that both high ratio of T790M sensitizing mutation (sensi‐mutation) and high MAFs of mutations were all associated with better third generation TKI treatment outcomes.

The quantification of both MAFs and T790M sensi‐mutation ratio should be taken into consideration in some clinical situations, and incorporation of high‐throughput NGS into clinical trials may be crucial to identifying the response to osimertinib, as it provides more comprehensive genomic information.

## Introduction

Lung cancer represents the leading cause of cancer‐related deaths worldwide.^1^ A subgroup of non‐small lung cancer (NSCLC) patients have been reported to have activating *EGFR* mutations,^2^ and responded well to first‐generation *EGFR* tyrosine kinase inhibitors (*EGFR*‐TKIs). However, most patients harboring EGFR‐activating mutations eventually acquire resistance to the first‐generation TKI treatment after a median response of 10–12 months.^3^ The most common resistance mechanism is the development of T790M mutation in exon 20 of the *EGFR* gene.^3^ *MET* amplification, *ERBB2* amplification, *PIK3CA* mutation and *BRAF* mutations have also been reported to cause resistance.^4^, ^5^

Unfortunately, clinical detection of resistance mechanism through tissue biopsy can be really challenging due to risks associated with the procedure, particularly for TKI‐relapsed patients. Tissue biopsy can also be incomplete because of spatial and temporal tumor heterogeneity. Liquid biopsy obtained by minimal invasive blood draws are therefore became accessible at almost all clinical situations.^6^ The fraction of circulating tumor DNA (ctDNA) in cell‐free DNA (cfDNA) varies according to tumor stage, tumor burden, vascularization of the tumor, apoptotic rate of tumor and the metastatic potential of the cancer cell.^7^ As a result, ctDNA often represents a small percentage of the total cfDNA and can be present at really low allele fractions. Therefore, highly sensitive methodologies should be used to detected low abundance mutations from cfDNA in NSCLC patients.^8^, ^9^ Currently, digital PCR, BEAMing (beads, emulsion, amplification and magnetics), and next‐generation sequencing (NGS) are widely used in genetic testing of ctDNA.^10^ Although some of these methods enable a sensitivity detection down to 0.01%, only NGS utilizes parallel sequencing to detect a broader range of genomic alterations by multitarget gene panels.

In this retrospective study, clinical validation of liquid biopsy was performed by concordance, sensitivity, and specificity of ctDNA hybrid capture panel NGS using 39 paired plasma and tissue biopsy from lung cancer patients. We also investigated the resistance mechanisms of 147 plasma samples from first‐generation TKI‐relapsed patients, and analyzed the ability to predict target therapy outcomes by ctDNA NGS.

## Methods

### Patients

We retrospectively reviewed all the patients who had been diagnosed with NSCLC and undergone ctDNA genetic test using NGS between March 2017 to May 2018 at the Cancer Hospital, Chinese Academy of Medical Sciences (CAMS, Beijing, China). A total of 39 blood samples with paired tissue biopsy samples were enrolled in our study for the comparison between liquid and tissue biopsy. Tissue biopsy samples were all collected at the same time period with blood samples. Another 147 blood samples from first‐generation *EGFR*‐TKI‐relapsed patients were also enrolled in the study in order to analyze the ability to predict target therapy outcomes by ctDNA NGS.

### Study design

**Figure 1:**
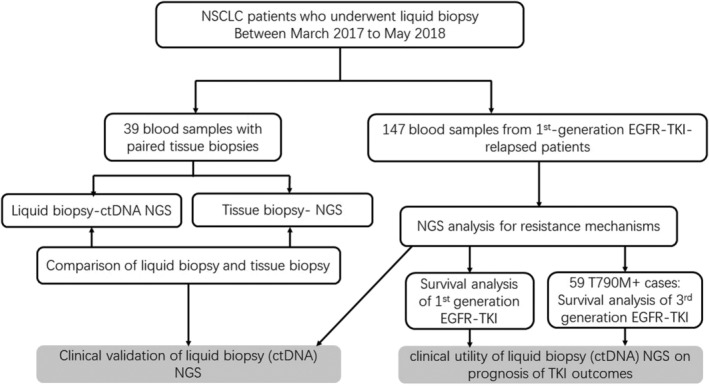
Study flow chart. Abbreviations: ctDNA, circulation tumor DNA; NGS, next generation sequencing; NSCLC, non‐small cell lung cancer.

The study included 39 blood samples with paired tissue biopsy samples and 147 blood samples collected from first‐generation *EGFR*‐TKI‐relapsed patients (Fig 1). Clinicopathological parameters were collected through electronic medical records. All tissue samples were formalin‐fixed paraffin‐embedded. For survival analysis, 147 TKI‐relapsed patients were followed up since their first dose of TKI until disease progression. This study was approved by the institutional review board of the Cancer Hospital, CAMS, and was conducted in accordance with the Declaration of Helsinki.

### FFPE tissue specimen processing and DNA extraction

Tissue biopsy samples were collected and processed by standard procedures. DNAs from FFPE samples were extracted using QIAamp DNA FFPE Tissue Kits (Qiagen, Duesseldorf, Germany), following the manufacturer's instructions. DNA quantity was determined by Qubit 2.0 Fluorometer (Thermo Fisher Scientific, Carlsbad, CA, USA).

### Plasma processing and DNA extraction

We collected 10 mL of whole blood into EDTA‐containing tubes, and plasma was separated within one hour of collection. Briefly, whole blood at 4°C was centrifuged at 2000 × g for 10 minutes, and the supernatant was transferred to a new 15 mL tube and centrifuged at 16000 × g for another 10 minutes at 4°C. About 4 mL plasma was then collected for DNA extraction. CtDNA was extracted from 4 mL plasma using the Plasma Circulating Nucleic Acid Preparation Kit (Qiagen, Duesseldorf, Germany), following the manufacturer's instructions. DNA quantity was determined by Qubit 2.0 Fluorometer (Thermo Fisher Scientific, Carlsbad, CA, USA).

### NGS analyses

Targeted NGS were performed using a capture‐based panel that covers 168 cancer‐related genes as previously reported.^11^ Available indexed samples were sequenced on NextSeq (Illumina, San Diego, CA) with paired‐end reads. The minimal median sequencing depth was 12 000× for ctDNA and 1000× for tissue DNA.

### Clinical response evaluation

The response evaluation was assessed according to Response Evaluation Criteria in Solid Tumors (RECIST), version 1.1^12^ by an independent medical oncologist. Progression‐free survival (PFS) was calculated from the date of initiation of TKI to the date of disease progression. The objective response rate (ORR) was calculated as the total percentage of patients with optimal efficacy of complete response (CR) or partial response (PR). The disease control rate (DCR) was calculated as the total percentage of patients with CR, PR or stable disease (SD).

### Statistical analysis

All statistical analyses were performed using the Statistical Package for the Social Sciences (SPSS, Chicago, IL, USA) version 22.0. Differences between groups were compared using the Pearson's χ^2^ test for categorical data, and *t*‐tests for continuous data. PFS were estimated using the Kaplan‐Meier method, and differences in PFS were assessed by log‐rank test. A two‐sided *P*‐value <0.05 was considered statistically significant.

## Results

### Comparison of liquid and tissue biopsy

A total of 39 paired blood and tissue biopsy were enrolled in our study. Nine paired tissue‐liquid samples were obtained from the TKI‐resistant patients, while the other 30 paired samples were obtained from TKI‐naïve patients. NGS tests using ctDNA revealed 15 cases were negative for all driver‐gene alterations, 12 cases harboring *EGFR* mutations, three cases harboring *KRAS* mutations, seven cases harboring *ALK* rearrangement, one case with *ROS1* rearrangement, and one case with *RET* rearrangement. High concordance of 84.62% were observed between ctDNA and tissue biopsies (Table 1). NGS sensitivity for ctDNA was 82.14% and specificity was 90.91%. It should be noted that the case with *RET* rearrangement in ctDNA NGS showed negative results in its paired tissue biopsy NGS. The probable reason for this was tumor heterogeneity.

**Table 1:** Comparison of ctDNA NGS and tissue biopsies NGS (*N* = 39)

		Tissue biopsy NGS		
Tissue biopsy Liquid biopsy		Negative	Positive	Concordance
ctDNA NGS	Negative	10†	5‡	84.62%††
	Positive	1§	23¶	

† Including two stage I–II cases and 8 stage III–IV cases.

‡ Including two stage I–II cases and 3 stage III–IV cases.

§ Stage III–IV patients.

¶ Including one stage I–II cases and 22 stage III–IV cases.

†† The concordance was 88.24% for late stage (stage III–IV) patients.

Among nine TKI‐resistant paired samples, two cases were proved to be ctDNA NGS false negative with positive tissue NGS results (one gefitinib‐resistant case and one crizotinib‐resistant case). Among 30 TKI‐naïve paired samples, four cases were proved to be ctDNA NGS false negative. For the type of biopsy procedure,, 21 patients underwent lung biopsies (13 transbronchial lung biopsies, and nine transthoracic needle lung biopsies), six cases obtained tumor cells from malignant pleural effusion, and eight cases underwent cervical lymph node biopsies. Liver, hip, and brain biopsies were used for the other three cases, respectively.

Among these 39 patients, five were early stage patients (I–II stage) and 34 were late stage patients (III–IV stage) (Table 1). If we only took late stage patients into consideration, the concordance, sensitivity, and specificity for ctDNA among late stage patients were 88.24%, 88.00% and 88.89%, respectively.

### Patient characteristics

The clinicopathologic characteristics of 147 *EGFR*‐TKI‐relapsed patients are summarized in Table 2. The median age at diagnosis was 62 years (range 34–83) and 57.14% (84/147) patients were female. A total of 59.18% (87/147) of patients were never smokers, and 21.77% (32/147) were former smokers (with 28 cases missing data). A total of 57.14% (84/147) were stage IIIB/IV patients without surgery, while 41.50% (61/147) were postoperative recurrent patients. The most common *EGFR* mutation type was L858R (44.9%, 66/147), followed by exon19 deletions (19del) (40.8%, 60/147). Other clinical characteristics such as metastasis status are listed in Table 2.

**Table 2:** Clinicopathological characteristics of *EGFR*‐TKI‐relapsed patients

Characteristics	No. of patients (*N* = 147)
Median age, years (range)	62 (34–83)
Sex	
Male	63 (42.86%)
Female	84 (57.14%)
Tobacco	
Ever	32 (21.77%)
Never	87 (59.18%)
Missing data	28 (19.05%)
Stage before TKI treatment	
IIIB/IV without surgery	84 (57.14%)
Postoperative recurrent	61 (41.50%)
Missing data	2 (1.36%)
Sensitizing mutation before TKI treatment	
18 exon (G719X)	5 (3.40%)
18 + 20 exon (G719X + L861X)	4 (2.72%)
20 exon alterations (20ins or S768X)	3 (2.04%)
19 exon deletions (19del)	60 (40.82%)
L858R	66 (44.89%)
Missing data	9 (6.12%)
Metastases (when liquid biopsy was performed)	
M0/M1a	52 (35.37%)
M1b	91 (61.90%)
Missing data	4 (2.72%)
Bone metastases	
No	88 (59.86%)
Yes	55 (37.41%)
Missing data	4 (2.72%)
Brain metastases	
No	68 (46.26%)
Yes	56 (38.10%)
Missing data	23 (15.65%)

### NGS results for *EGFR*‐TKI‐relapsed patients

**Figure 2:**
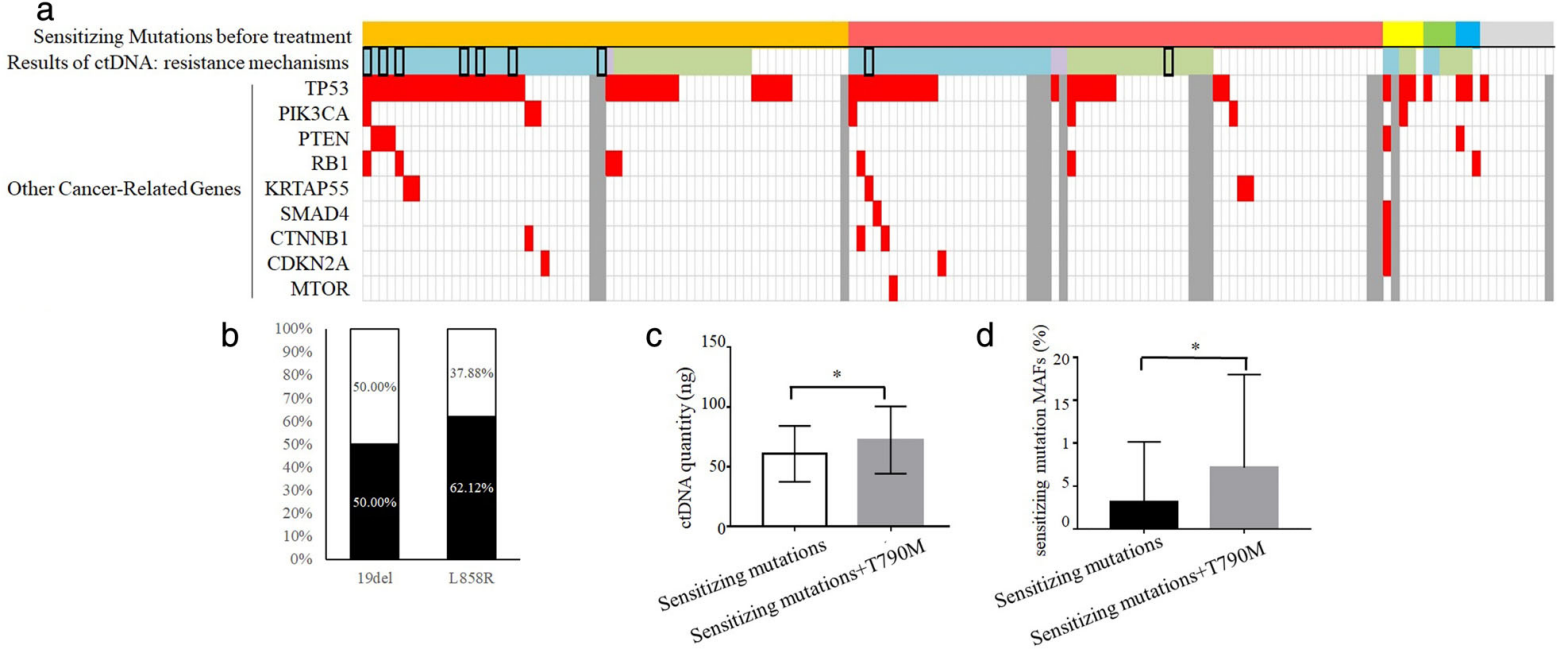
NGS results for ctDNA from 147 first‐generation *EGFR*‐TKI‐relapsed patients. (**a**) Sensitizing mutation types before TKI treatment of 147 cases, and mutation profiling of ctDNA NGS results. (**b**) Comparison of T790M frequency between different sensitizing mutation types (19del vs. L858R). (**c**) Difference in ctDNA quantity between T790M‐positive patients and patients only been detected with sensitizing mutations. (**d**) Difference in sensitizing mutation MAFs between T790M‐positive patients and patients only been detected with sensitizing mutations. (**a**) 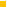 19DEL, 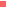 L858R, 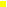 18exon, 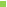 18+20exons, 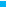 20exon, 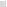 unknown sensitizing mutations, 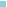 sensitizing mutations+T790M, 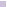 sensitizing mutations+alternative pathway activations, 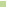 only sensitizing mutations, 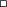 with *EGFR* amplification, 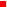 mutations, 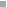, Not Available, (**b**) 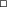 T790M1+, 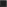 T790M1−.

Cancer‐related gene mutations were detected in most patients (81.63%, 120/147) including *EGFR*, *MET*, *ERBB2*, *TP53*, *PIK3CA*, *PTEN*, *RB1*, *SMAD4*, *CTNNB1*, *CDKN2A*, and *MTOR*, whereas 27 patients (18.36%) were negative in the NGS test (Fig 2a). T790M was observed as resistance mechanism in 40.13% (59/147) patients; *EGFR* sensitizing mutations without T790M were only detected in 27.89% (41/147) of patients; and 29.93% (44/147) of patients were negative for *EGFR* mutations (Fig 2a). For the 44 patients, the median age at diagnosis was 62.5 years (range 40–81) and 56.82% (25/44) patients were female. A total of 47.73% (21/44) received *EGFR*‐TKI treatment as first‐line, while 50.00% (22/44) received *EGFR*‐TKI after chemotherapy (one missing data). A total of 36.36% (16/44) of those patients had brain metastases when liquid biopsy was performed. No difference in brain metastases ratio were observed between the 44 patients and others with *EGFR* mutations. However, bone metastases were more common in patients detected with *EGFR* mutations than those without any *EGFR* mutations (44.00% vs. 25.58%, *P* = 0.042). No other significant difference of clinical characteristics were observed between patients who were negative for *EGFR* mutations and others. We also found 2.04% (3/147) of patients developed alternative pathway activations (including *MET* amplification and *ERBB2* amplification) as resistance mechanisms. A total of 133 cases have been tested for *TP53* status, and 45.86% (61/133) of them detected with *TP53* mutations. *TP53* mutations were more common observed with a concurrent 19del mutation than L858R (*P* = 0.014, Fig S4a). We also found that *TP53* mutations much more frequently coexisted with other cancer‐related gene alterations (*P* = 0.025, Fig S4b).

T790M was more likely to coexist with 19del than L858R, although with a borderline *P*‐value (Fig 2b, *P* = 0.171). Compared with cases only detected with sensitizing mutations, the T790M‐positive group seemed to have higher ctDNA quantity for NGS library construction (mean ± SD: 72.37 ± 28.17ng vs. 60.82 ± 23.38 ng, *P* = 0.033, Fig 2c) and higher sensitizing mutant allele frequency (MAF) (mean MAF ± SD: 7.13% ± 10.88% vs. 3.13% ± 7.02%, *P* = 0.031, Fig 2d). In the T790M‐positive group, the ratio of T790M/sensitizing mutation in 80% patients was below 100%, which suggested intratumor heterogeneity of T790M.

Among 59 T790M‐positive cases, the association of clinical characteristics and MAFs of T790M were also analyzed (one missing data). A total of 35.59% (21/59) of patients underwent surgeries before TKI treatment. When ctDNA NGS was performed, 50.85% (30/59) had bone metastases, 37.29% (22/59) had brain metastases. The majority of patients (74.58%, 44/59) had extrapulmonary metastases (M1b), 18.64% (11/59) were M1a, while only three cases (5.08%) did not have metastasis. We found that the MAF of T790M were significantly higher in M1b cases than others (M0 and M1a) (mean ± SD: 4.66% ± 7.27% vs. 0.39% ± 0.39%, *P* < 0.001). No other significant association was observed between other clinical characteristics and T790M MAFs. No significant association was observed between clinical characteristics and sensi‐mutation MAFs.

### Survival analysis of first‐generation TKI

All 147 patients received first‐generation *EGFR*‐TKI treatment between March 2007 to Feb 2018, and were diagnosed with disease progression. ORR for first‐generation TKI was 54.47% and DCR was 96.27%. No significant difference of ORR or DCR was observed between 19del and L858R. However, the *TP53* mutation group had a much lower ORR than the *TP53* wild‐type group (*P* = 0.046, Table 3).

**Table 3:** Response to treatment for patients with *EGFR*‐TKI

	Sensitizing mutations			T790M status after first TKI relapsed			*TP53* status		
	19del	L858R	*P*	Positive	Negative	*P*	Positive	Negative	*P*
Response to first‐generation TKI									
Type of response ‐ No. (%)									
Complete response	1 (1.67)	2 (3.03)		1 (1.69)	2 (2.27)		2 (3.28)	0 (0)	
Partial response	27 (45.00)	34 (51.52)		24 (40.68)	46 (52.27)		23 (37.70)	42 (58.33)	
Stable disease	25 (41.67)	24 (36.36)		31 (52.54)	25 (28.41)		28 (45.90)	23 (31.94)	
Progressive disease	3 (5.00)	1 (1.52)		0 (0)	5 (5.68)		3 (4.92)	2 (2.78)	
Could not be evaluated	4 (6.67)	5 (7.58)		3 (5.08)	10 (11.36)		5 (8.20)	5 (6.94)	
Objective response rate ‐ % (95% CI)	50.00 (36.49–63.51)	59.02 (46.32–71.72)	0.332	44.64 (31.21–58.08)	61.54 (50.49–72.58)	0.053	44.64 (31.21–58.08)	63.69 (50.80–74.57)	**0.046**
Disease control rate % (95% CI)	94.64 (88.56–100)	98.36 (95.08–100)	0.284	100	93.59 (88.03–99.15)	0.054	95.64 (88.56–100)	97.01 (92.83–100)	0.511
Time to response ‐ months									
Mean	3.74	6.29	0.102	2.66	8.07	**0.000**	3.59	6.59	**0.033**
95% CI	2.08–5.40	3.78–8.81		1.92–3.40	5.51–10.64		1.97–5.21	4.32–8.86	
Duration of response – months									
Mean	13.30	15.95	0.490	16.57	11.49	0.136	14.35	12.78	0.651
95% CI	8.33–18.27	10.09–21.81		11.57–21.57	7.07–15.90		8.65–20.06	8.58–16.98	
Response to third‐generation TKI									
Type of response ‐ No. (%)									
Complete response	0 (0)	0 (0)		0 (0)	‐	‐	0 (0)	0 (0)	
Partial response	17 (56.67)	7 (28.00)		26 (44.07)	‐	‐	17 (51.52)	7 (35.00)	
Stable disease	7 (23.33)	13 (52.00)		20 (33.90)	‐	‐	9 (27.27)	8 (40.00)	
Progressive disease	0 (0)	0 (0)		1 (1.69)	‐	‐	1 (3.03)	0 (0)	
Could not be evaluated	6 (20.00)	5 (20.00)		12 (20.34)	‐	‐	6 (18.18)	5 (25.00)	
Objective response rate ‐ % (95% CI)	70.83 (51.23–90.44)	35.00 (12.10–57.90)	**0.017**	55.32 (40.56–70.07)	‐	‐	62.96 (43.50–82.43)	46.67 (18.07–75.26)	0.318
Disease control rate ‐ % (95% CI)	100	100	‐	97.87 (93.59–100)	‐	‐	96.30 (88.68–100)	100	0.463
Time to response ‐ months									
Mean	1.96	1.79	0.697	1.92	‐	‐	1.77	1.95	0.708
95% CI	1.41–2.51	1.03–2.54		1.48–2.34	‐		1.16–2.38	1.19–2.70	
Duration of response ‐ months									
Mean	4.98	6.07	0.447	5.66	‐	‐	4.54	7.16	0.092
95% CI	2.65–7.31	3.85–8.28		4.25–7.06	‐		2.40–6.68	4.39–9.94	

The median PFS of first‐generation TKI was 13.73 months (95% CI: 12.16–15.31 months, Fig S1a). Sensitizing mutation types showed no significant influence on PFS, neither did *TP53* status (Fig S1b,S1c). The T790M‐positive group had a much shorter time to response than the T790M‐negative group (*P* = 0.000, Table 3). Also, the *TP53* positive group had a shorter time to response than the *TP53* wild‐type group (*P* = 0.037, Table 3).

### Survival analysis of third‐generation TKI

**Figure 3:**
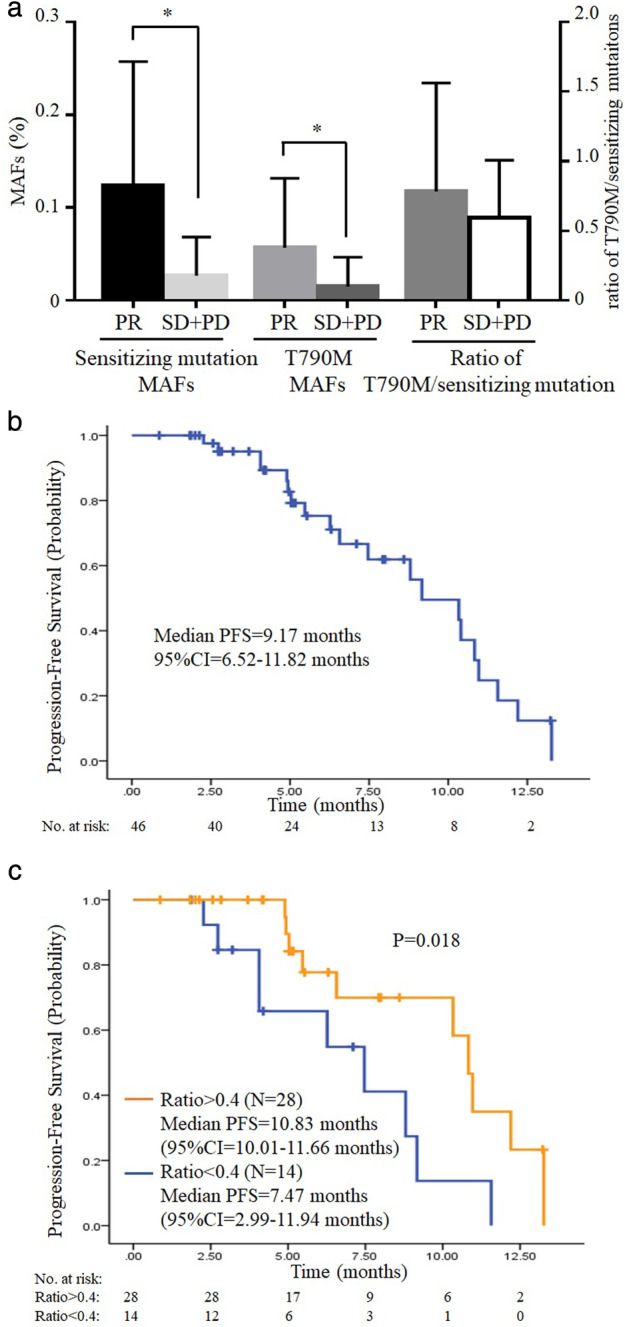
Survival analysis of third‐generation TKI. (**a**) Comparison of sensitizing mutation MAFs, T790M MAFs and ratio of T790M sensitizing mutation (sensi‐mutation) according to optimal efficacy of third‐generation TKI. (**b**) Kaplan‐Meier estimates of third‐generation TKI progression‐free survival (PFS). (**c**) Survival curves according to ratio of T790M sensi‐mutation.

Among 59 patients detected with T790M, 46 patients had received osimertinib treatment during September 2015 to June 2018. The last follow‐up date was 31 August 2018, when 43.48% (20/46) of patients progressed from third‐generation TKI treatment. A total of 41.30% (19/46) of these patients were treated with second‐line therapy, and 43.48% (20/46) of patients received osimertinib as third‐line treatment (chemotherapy as first‐line therapy, and first‐generation TKI as second‐line therapy). Six patients received more than two lines of treatment before osimertinib, with one missing data. ORR and DCR for third‐generation TKI were 55.32% and 97.87%, respectively. Patients harboring 19del had higher ORR than those harboring L858R (*P* = 0.017, Table 3). Moreover, compared with optimal efficacy of the SD + PD group, the PR group seemed to have higher MAF of sensitizing mutation (mean ± SD: 12.37% ± 13.37% vs. 2.67% ± 4.17%, *P* = 0.002) and higher T790M MAF (mean ± SD: 5.68% ± 7.47% vs. 1.49% ± 3.18%, *P* = 0.014) (Fig 3a).

The median PFS of third‐generation TKI was 9.17 months (95% CI = 6.52–11.82 months, Fig 3b). No significant difference of PFS was observed between the 19del and L858R groups, nor *TP53* status (Fig S2a,S2b). A receiver operating characteristic curve (ROC curve) was drawn to determine the most appropriate cutoff value of T790M sensitizing mutation (sensi‐mutation) ratio for predicting third‐generation TKI progression (Fig S3). According to ROC curve, we chose ratio = 0.4 (point *a* in Fig S3) as the cutoff value, and categorized all these patients into two groups: ratio > 0.4 group (28 cases) and ratio < 0.4 group (14 cases). Using Kaplan‐Meier analysis, ratio > 0.4 group had a much better PFS of third‐generation TKI than ratio < 0.4 group (*P* = 0.018, Fig 3(c)).

## Discussion

Molecular testing using high throughput NGS can provide more comprehensive genomic information for detection of resistance mechanisms and treatment outcome monitoring.^13^ Although the gold standard to assess genomic mutation is through the detection of tissue biopsy, tumor tissue is not always available due to the limitation of invasive procedures, insufficient sample for testing, and intratumor heterogeneity. Liquid biopsy of ctDNA has proven to be an adequate source for biomarker testing in targeted therapy. However, there are few studies reporting the clinical utility of liquid biopsy in the real world. Our study was aimed at clinical validation of liquid biopsy NGS by comparison with tissue biopsy, and also investigated clinical utility of ctDNA NGS on prediction of third‐generation TKI outcomes in a large Chinese cohort.

In our study, concordance for ctDNA and tissue biopsy was 84.62% among all stage patients, and even higher among late stage patients (88.24%), consistent with previous studies.^14^, ^15^ We also investigated the resistance mechanisms of 147 plasma from first‐generation TKI‐relapsed patients, and T790M was the most common reason for the resistance of first‐generation TKI (40.13%) (Fig 2a). Taniguchi *et al*. also reported that T790M occurred in 43.5% of TKI‐relapsed patients using ctDNA BEAMing.^16^ Our results indicated that NGS‐based platform is competent for detection of ctDNA mutations to guide the target therapy, especially when resistance to TKI occurs and no tumor tissue is available.

We found that T790M was more likely to coexist with 19del than with L858R (50% vs. 37.88%, Fig 2b). Ke *et al*. reported that prevalence of T790M was significantly higher in 19del than in the L858R subgroup (50.4% vs. 36.4%, *P* = 0.043).^17^ A previous in vitro and in vivo study proved that 19del/T790M cells had increased oncogenic activity compared with L858R/T790M cells.^18^ Therefore, we speculate that 19del cell clones may have the ability to make T790M become the dominant cell clones after TKI treatment. In the study by Babu Koyyala *et al*. the incidence of T790M were similar between the 19del and L858R group (55.56% vs. 53.85%)^19^; while Del Re *et al*. proved that the incidence of T790M in 19del was slightly lower than in L858R after gefitinib/erlotinib treatment (73.08% vs. 84.62%), but T790M was more likely to coexist with 19del than L858R after afatinib treatment (38.24% vs. 14.29%).^20^ We believe that the different technology used and different population enrolled in the studies could both influence the results. In addition, although different opinions have been reported, few of these studies had a statistically significance conclusion. Therefore further studies with a larger population are still needed to confirm the results.

In our study, the ratio of T790M sensi‐mutation in most patients was below 100%, which indicated that T790M was present in a subpopulation of *EGFR* sensitizing mutation cells. Interestingly, we also found that compared with those detected with both sensitizing mutations and T790M, patients only detected with sensitizing mutations had a lower MAF of sensitizing mutations (Fig 2d). We also found that T790M MAFs were significantly associated with metastasis status, and M1b patients had higher T790M MAFs than others. It should be noted that for those patients without extrapulmonary metastases, it is likely that T790M in ctDNA was too low to be detected. These findings highlight the possibility that some patients might have T790M MAF lower than the limitation of detection (LOD) and only sensitizing mutations were detected. It is a reminder that for patients harboring only sensitizing mutation with low MAF in ctDNA test, there is possibility that they can still benefit from third‐generation TKI treatment.

In survival analysis of first‐generation TKI, the median PFS was 13.73 months, consistent with the published data.^21^ ORR and DCR for first‐generation TKI were 54.47% and 96.27%, respectively. Although no significant influence was observed on PFS for sensitizing mutation types and *TP53* status, we did find that *TP53* mutation patients had a much lower ORR than *TP53* wild‐type patients. A previous study also reported that *TP53* missense mutations was significantly associated with DCR for first‐generation TKI,^22^ which also indicated the negative impact of *TP53* mutation on target therapy.

In survival analysis of third‐generation TKI, the median PFS was 9.17 months. ORR and DCR for third‐generation TKI were 55.32% and 97.87%, respectively, which was consistent with the previous report.^6^ It should be noted that in our study, higher MAFs of both sensitizing mutation and T790M were associated with better optimal efficacy (Fig 3a). Recently, Li *et al*. reported that the abundance of *EGFR* sensitizing mutations was significantly associated with ORR to first‐generation TKIs,^23^ and also a Japanese study reported L858R MAF may be a potential predictive factor of TKI treatment efficacy.^24^ We inferred that low MAFs of both sensitizing mutations and T790M caused by the intratumor heterogeneity may contribute to resistance of TKI treatment and played a negative role in targeted treatment efficacy. Using Kaplan‐Meier analysis, we found that the high ratio of T790M/sensitizing mutation (ratio > 0.4) group benefited longer PFS of third‐generation TKI (Fig 3c). Similar results were reported by Ariyasu *et al*. among 33 tumor tissues from first‐generation TKI‐relapsed patients.^25^ Marzia Del Re *et al*. reported a different cutoff ratio for T790M sensi‐mutation. In their study, cfDNA was analyzed by droplet digital PCR (ddPCR), and patients with T790M sensi‐mutation >0.22 had better PFS than others.^26^ We believe that the different technology used in their research might be the reason for the difference between cutoff ratios. We used capture‐based NGS for cfDNA analysis, which might have a lower efficiency in capture deletions than ddPCR.^27^ Since there were 47.83% (22/46) patients who received osimertinib harboring 19del in our study, it is reasonable that T790M sensi‐mutation ratio was higher than the ratio in the study by Del Re *et al*. Thus, the cutoff ratio in our study was slightly higher than the study by Del Re *et al*. As we revealed that the ratio of T790M/sensitizing mutation varied in different patients because of intratumor heterogeneity, the most possible reason for the negative impact of low T790M ratio on third‐generation TKI PFS is that clones lacking T790M are likely to have other resistance mechanisms and respond poorly to third‐generation TKI treatment.

Our study was limited by a few factors. First, our work was a retrospective study and only included first‐generation TKI‐relapsed blood samples. Baseline blood samples collected before first‐generation TKI treatment may be more effective to predict first‐generation TKI outcomes. Second, the number of T790M‐positive patients received osimertinib was relatively small, and not all progressed from third‐generation TKI treatment. Future studies are still needed to confirm the results with a larger population.

In summary, our study indicated that high‐throughput NGS based platforms are competent for the detection of ctDNA mutations to guide the target therapy, especially when tissue biopsy was not available. However, it should always be noted that for those patients harboring low MAF sensitizing mutation without T790M in ctDNA NGS, it is possible that their T790M mutation is under LOD, and can still benefit from third‐generation TKI treatment. In survival analysis, *TP53* mutation showed negative impact on target therapy outcomes. We also found that in third‐generation TKI treatment, both high ratio of T790M sensi‐mutations and high MAFs of *EGFR* mutations were associated with better outcomes of third‐generation TKI treatment. Therefore, the quantification of both MAFs and T790M sensi‐mutation ratio should be taken into consideration in some clinical situations, and incorporation of high‐throughput NGS into clinical trials may be crucial in identifying the response to osimertinib, as it provides more comprehensive genomic information, such as mutation types and the MAF.

## Disclosure

No authors report any conflict of interest.

## Supporting information


**Figure S1** Kaplan‐Meier estimates of first‐generation TKI progression‐free survival of 147 cases. (**a**) Survival curves of all 147 cases; (**b**) survival curves according to sensitizing mutation types; and (**c**) survival curves according to TP53 status.
**Figure S2** Kaplan‐Meier estimates of third‐generation TKI progression‐free survival of 46 patients. (**a**) Survival curves according to sensitizing mutation types; and (**b**) survival curves according to TP53 status.
**Figure S3** Receiver operating characteristic (ROC) curve, point *a*: ratio (T790M sensitizing mutation) = 0.4.
**Figure S4** The association between *TP53* status and other genetic alterations. (**a**) *TP53* status and EGFR sensitizing mutations. (**b**) *TP53* status and other cancer‐related gene mutations detected by next‐generation sequencing (NGS).
