# Supplementary material for: High MAF of EGFR mutations and high ratio of T790M sensitizing mutations in ctDNA predict better third‐generation TKI outcomes
Source: Thorac Cancer. 2020 Apr 14;11(6):1503–11. doi: 10.1111/1759-7714.13418 (PMC7262937; doi:10.1111/1759-7714.13418)

Supplementary Figure S1. Kaplan-Meier estimates of first-generation TKI progression-free survival of 147 cases. A). Survival curves of all 147 cases; B). Survival curves according to sensitizing mutation types; C). Survival curves according to TP53 status.


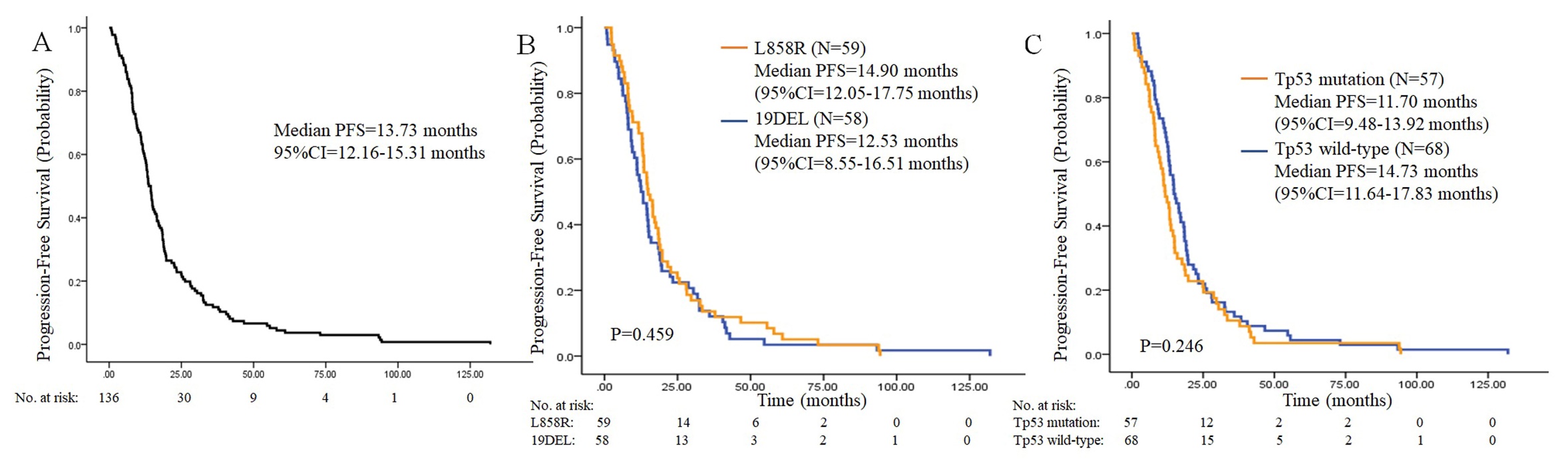


Supplementary Figure S2. Kaplan-Meier estimates of third-generation TKI progression-free survival of 46 patients. A). Survival curves according to sensitizing mutation types. B, Survival curves according to TP53 status.


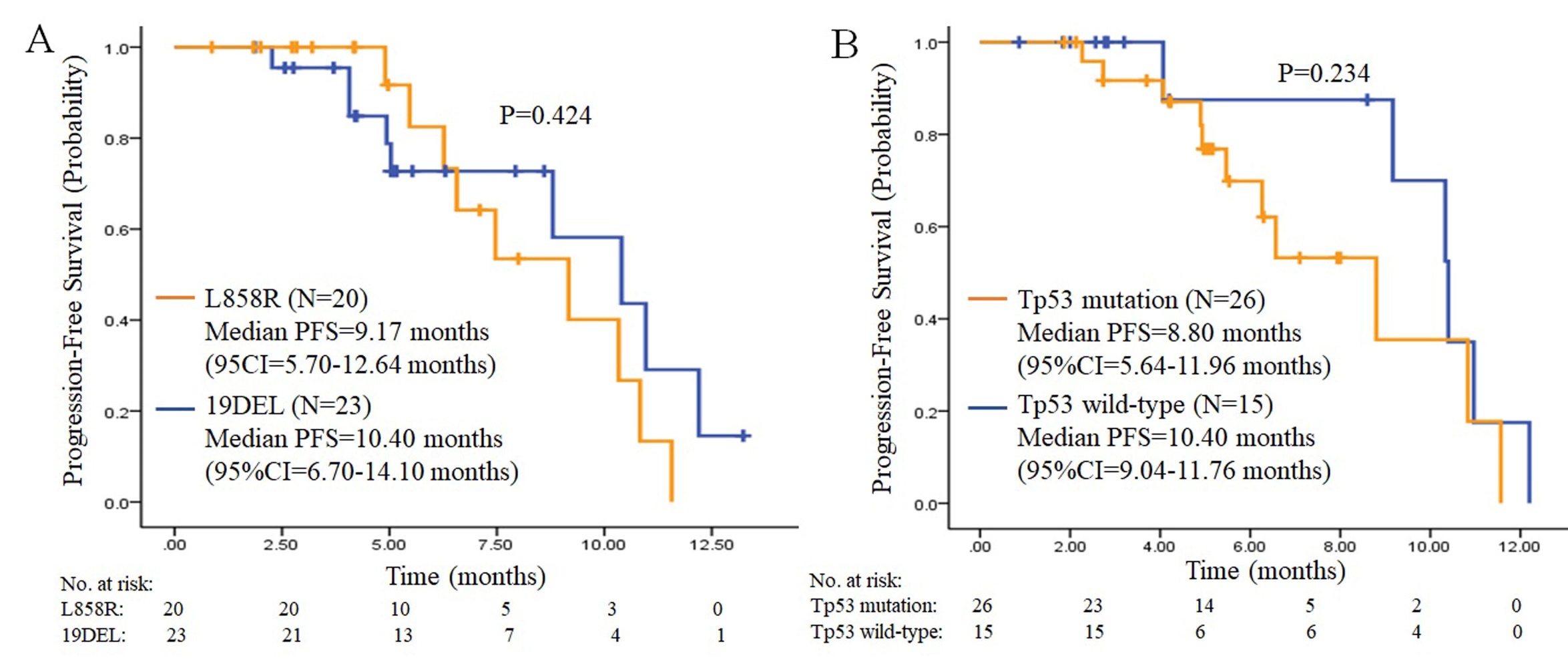


Supplementary Figure S3. Receiver operating characteristic (ROC) curve, point *a*: ratio (T790M/sensitizing mutation) = 0.4.


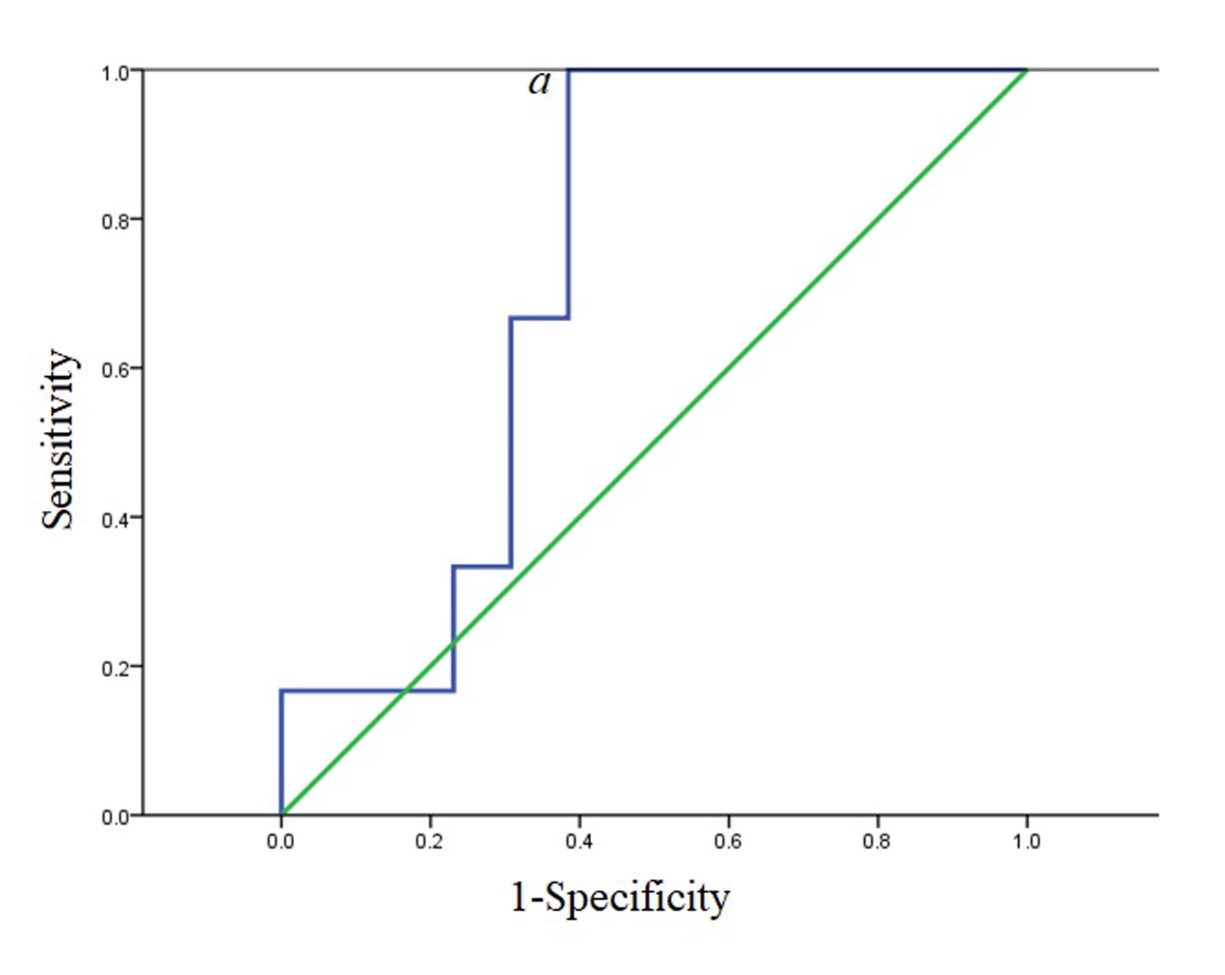


Supplementary Figure S4. The association between *TP53* status and other genetic alterations. A). *TP53* status and EGFR sensi-mutations. B). *TP53* status and other cancer-related gene mutations detected by NGS.


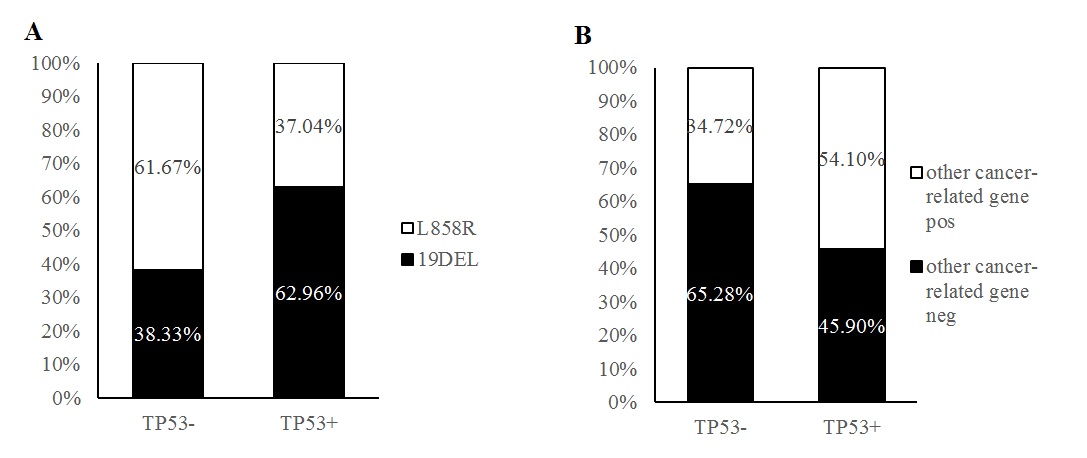

Supplement: Supplementary file 1 — Figure S1 Kaplan‐Meier estimates of first‐generation TKI progression‐free survival of 147 cases. (a) Survival curves of all 147 cases; (b) survival curves according to sensitizing mutation types; and (c) survival curves according to TP53 status. Figure S2 Kaplan‐Meier estimates of third‐generation TKI progression‐free survival of 46 patients. (a) Survival curves according to sensitizing mutation types; and (b) survival curves according to TP53 status. Figure S3 Receiver operating characteristic (ROC) curve, point a: ratio (T790M sensitizing mutation) = 0.4. Figure S4 The association between TP53 status and other genetic alterations. (a) TP53 status and EGFR sensitizing mutations. (b) TP53 status and other cancer‐related gene mutations detected by next‐generation sequencing (NGS). [file TCA-11-1503-s001.docx]
